# Role of Adenosine/ATP Test in Supraventricular Tachycardia

**Published:** 2003-01-01

**Authors:** Johnson Francis

**Affiliations:** Associate Professor of Cardiology, Medical College Calicut, Kerala, India

The successful treatment of paroxysmal supraventricular tachycardia with adenosine 5` triphosphate (ATP) was initially reported by Somlo [1] in 1955. Sharma et al [2] noted the value of intravenous ATP in the diagnosis and management of wide QRS complex tachycardia. ATP in incremental doses has been used for the non-invasive diagnosis of concealed accessory pathways [3].

Non-invasive diagnosis of dual AV node physiology (DAVNP) in patients with AV nodal reentrant tachycardia by administration of ATP or adenosine has been reported by different authors [4,5]. Though many of the studies were using incremental dosage, single dose tests with ATP [6] and adenosine [7] have also been useful in the diagnosis of DAVNP. The test is useful in sinus rhythm to establish the presence of DAVNP. DAVNP is considered to be present when at least one of the following events occur following ATP injection: 1) PR interval increases or decreases by >50 ms in 2 consecutive sinus beats; 2) an AV nodal echo beat is observed; 3) AVNRT develops. In this issue of the Journal, Belhassen et al [8] report that DAVNP is present in a relatively high proportion (36.5%) of patients following termination of AVNRT with ATP but is much less frequent (5.5%) in control patients. In addition, they show that the occurrence of DAVNP following the administration of ATP in sinus rhythm is a good predictor of its occurrence after termination of AVNRT with ATP. Thus, findings at termination of tachycardia by ATP may be useful in the noninvasive diagnosis of the mechanism of a paroxysmal supraventricular tachycardia.

In patients with palpitations of unclear etiology, ATP test identifies those who are likely to have AVNRT or AVRT (and who are therefore likely to benefit from electrophysiological evaluation) with a high positive predictive value [9]. ATP/adenosine test is also useful in confirming the result of radiofrequency ablation of slow pathway [6,10]. Among patients with no history suggestive of AVNRT, less than 3% have clinically silent DAVNP on incremental adenosine infusion [11]. Adenosine/ATP infusion can be used a simple bedside screening test for patients with symptoms suggestive of of paroxysmal supraventricular tachycardia which is undocumented and in those in whom the mechanism of a narrow complex tachycardia is unclear. This test may be specially relevant in developing countries with limited resources, in selecting patients for further electrophysiological evaluation.
